# The A226D Mutation of OmpC Leads to Increased Susceptibility to β-Lactam Antibiotics in *Escherichia coli*

**DOI:** 10.3390/biology13080600

**Published:** 2024-08-09

**Authors:** Jiaming Zhu, Peng Guo, Yuting Zheng, Shiqing Xiang, Yang Zhao, Xinyu Liu, Chengzhang Fu, Youming Zhang, Hai Xu, Ling Li, Wenjia Wang, Mingyu Wang

**Affiliations:** 1 State Key Laboratory of Microbial Technology, Microbial Technology Institute, Shandong University, Qingdao 266237, China; zhjm0121@163.com (J.Z.); guopeng21@mail.sdu.edu.cn (P.G.); tingzhengy@163.com (Y.Z.); sdushiqingx@163.com (S.X.); 202100141076@mail.sdu.edu.cn (Y.Z.); liuxy7717@163.com (X.L.); haixu@sdu.edu.cn (H.X.); lingli@sdu.edu.cn (L.L.); 2 School of Life Sciences, Shandong University, Qingdao 266237, China; 3 Helmholtz Institute for Pharmaceutical Research Saarland (HIPS), Helmholtz Centre for Infection Research (HZI), Department of Pharmacy, Saarland University, 66123 Saarbrücken, Germany; chengzhang.fu@helmholtz-hips.de

**Keywords:** antimicrobial resistance, outer membrane porin, β-lactam, OmpC, molecular dynamics

## Abstract

**Simple Summary:**

Antimicrobial resistance (AMR) is a problem of worldwide concern that has led to over one million deaths annually and can potentially lead to millions more in the future. Understanding mechanisms of AMR is of critical importance in the surveillance and treatment of antibiotic-resistant infections. Outer membrane porins (OMPs) are membrane-bound tunnels that are involved in the uptake of various chemicals into the cell. Previous research has shown that the absence of these proteins can lead to resistance to various antibiotics, but whether their mutations can play a role in antibiotic susceptibility has not been well characterized. This work reports an A226D mutation in OmpC in *E. coli* that is widespread in human-related strains, shows that this mutation led to increased susceptibility to β-lactam antibiotics, and proposes mechanisms underlying this phenotype with molecular dynamics. This work reports a new factor for modulating antibiotic susceptibility and improves our understanding of mechanisms of antibiotic resistance.

**Abstract:**

Bacterial resistance to antibiotics can lead to long-lasting, hard-to-cure infections that pose significant threats to human health. One key mechanism of antimicrobial resistance (AMR) is to reduce the antibiotic permeation of cellular membranes. For instance, the lack of outer membrane porins (OMPs) can lead to elevated AMR levels. However, knowledge on whether mutations of OMPs can also influence antibiotic susceptibility is limited. This work aims to address this question and identified an A226D mutation in OmpC, a trimeric OMP, in *Escherichia coli*. Surveillance studies found that this mutation is present in 50 *E. coli* strains for which whole genomic sequences are available. Measurement of minimum inhibition concentrations (MICs) found that this mutation leads to a 2-fold decrease in MICs for β-lactams ampicillin and piperacillin. Further survival assays confirmed the role this mutation plays in β-lactam susceptibility. With molecular dynamics, we found that the A226D mutation led to increased overall flexibility of the protein, thus facilitating antibiotic uptake, and that binding with piperacillin was weakened, leading to easier antibiotic penetration. This work reports a novel mutation that plays a role in antibiotic susceptibility, along with mechanistic studies, and further confirms the role of OMPs in bacterial tolerance to antibiotics.

## 1. Introduction

Antimicrobial resistance (AMR) ranks among the most concerning clinical issues of the 21st century, which results in the failure of antibiotic therapy, the primary tool to fight against bacterial infections. It is estimated that AMR led to 1.27 million deaths worldwide in 2019 [1]. To date, many mechanisms have been identified for bacterial AMR, including efflux pumps [2], modification or hydrolysis of antibiotic molecules [3], alteration of antibiotic targets [4], and reduction of antibiotic permeability [5].

Gram-negative bacteria possess a bilayer lipid membrane that serves as a protective barrier against various harmful substances, including antibiotics. However, antibiotics and other small molecules have the ability to penetrate cells via protein channels present on the cellular membrane that are responsible for material exchange, eventually leading to damage to bacteria [6]. Outer membrane porins (OMPs) are a class of channel proteins in Gram-negative bacteria, which are capable of facilitating antibiotic uptake [7]. These proteins are located in the outer membrane and create a tunnel that connects the outside of the cell and the periplasm [8]. The typical OMP is a trimeric protein, with each monomer generally consisting of a β-barrel made up of 16 β-strands [8]. The interaction between the monomers is reinforced by both hydrophobic and polar interactions [9]. Furthermore, the external surface of porin barrels is predominantly composed of lipophilic side chains, facilitating their positioning on the cell’s outer membrane [10].

Diffusion of small molecules including antibiotics into the cell is possible with OMPs. Therefore, OMPs are closely associated with the development of antibiotic resistance in bacteria. *Pseudomonas aeruginosa* and *Acinetobacter baumannii* exhibit intrinsic resistance to β-lactam antibiotics due to their low OMP permeability [11,12]. In addition, there are some bacteria whose OMPs have been shown to be the main gateway for antibiotics to enter cells [13,14]. The detection of *omp* genes is frequently conducted to explore the mechanism of multi-drug resistant (MDR) bacteria. The elevated frequency of detection and the presence of mutant proteins of OMP in MDR strains suggest a correlation between OMP and bacterial drug resistance [15,16,17,18].

*Escherichia coli* is an important commensal bacterium that is also an opportunistic pathogen. Three OMPs are present in *E. coli*, including OmpA, OmpC, and OmpF. Previous research has shown that upregulation of *ompC* leads to an increase in antimicrobial susceptibility [19], deletion or disruption of *ompC* can lead to increased AMR [20,21,22], and *ompC* levels were found to be correlated with AMR levels [23]. The study on whether OmpC mutation can impact antibiotic susceptibility, however, is limited. The Reference Gene Catalog of NCBI documented six mutations of OmpK36, a homolog of OmpC in *Klebsiella pneumoniae*, that moderated carbapenem resistance. No OmpC mutation influencing AMR levels was documented in this database.

In this work, an A226D mutation of OmpC was found in *E. coli*. Its influence on antimicrobial resistance was investigated in this work, and mechanisms for this influence were proposed based on computational analysis. This work demonstrates that mutations in OmpC can have a direct impact on AMR, which provides a new perspective for dealing with the AMR problem in the future. It is possible to design new drugs that selectively target OmpC or mutated OMP proteins at specific sites to mitigate bacterial resistance and combat antimicrobial resistance.

## 2. Materials and Methods

### 2.1. Search for E. coli Strains with OmpC A226D Mutation

To search for *E. coli* strains that encode OmpC A226D mutants, 36,431 *E. coli* whole genomic sequences were downloaded from GenBank. Prodigal was used to predict genes from whole genomic sequences [24], and Diamond was used to search for the OmpC (Uniprot: P06996) sequences in these genomes [25]. OmpC sequences with an A226D mutation were further picked for analysis of sources. The sources of *E. coli* strains were identified by checking for metadata on Genbank.

### 2.2. Mutation of ompC

The *ompC* gene in *E. coli* BW25113 and the pACYC-spt plasmid vector were amplified, followed by the ligation of the two fragments through homologous recombination. Subsequently, a plasmid containing the wildtype OmpC (pACYC-OmpC_WT_) was successfully constructed. The mutation codon was designed in the primer, and the wildtype plasmid pACYC-OmpC_WT_ was used as the template to introduce the A226D mutation into the plasmid through PCR amplification. The PCR reaction conditions included an annealing temperature of 55 °C, an amplification time of 3 min, and 30 amplification cycles. The MultiF Seamless Assembly Mix (Abclonal Technology Co., Ltd., Wuhan, China) was employed to ligate the amplified linearized plasmid fragment. Ultimately, the plasmid carrying the OmpC_A226D_ (pACYC-OmpC_A226D_) was successfully generated. Primers can be found in Table S1.

### 2.3. Construction of ompC-Containing Strains

The *E. coli* BW25113 Δ*ompC* strain was picked from the Keio *E. coli* knockout strain collection [26]. The constructed plasmids pACYC-OmpC_WT_ and pACYC-OmpC_A226D_ were further transformed into *E. coli* BW25113 Δ*ompC*, generating the *E. coli* OMP_WT_ and *E. coli* OMP_A226D_ strains. Primers can be found in Table S1.

### 2.4. Drug Susceptibility Test

Minimum inhibition concentrations (MICs) to ampicillin (AMP) and piperacillin (PIP) were determined with the broth dilution method following Clinical and Laboratory Standards Institute (CLSI) standards [27]. The highest antibiotic concentration used was 512 μg/mL, which was then subject to serial two-fold dilutions until it reached the lowest concentration of 0.125 μg/mL. The bacterial cultures were adjusted to a Mnemonic turbidity of 0.5 and subsequently diluted 100 times with Müller-Hinton Broth. Five microliters of bacterial culture was supplemented to 100 μL of diluted antibiotic solution. The absorbance of the resulting mixture was measured at 630 nm after a 16 h incubation period. The MIC value refers to the minimum antibiotic concentration that inhibits bacterial growth. *E. coli* ATCC 25922 was used as the quality control strain in this assay.

### 2.5. Survival Assays

Survival assays were performed to compare the tolerance of strains to AMP and PIP. Bacterial strains were grown to OD_600_ = 0.6, followed by AMP and PIP addition at final concentrations of 1, 4, 10, and 16 μg/mL. Cells were further grown by shaking at 37 °C for 1 h, and sampled at 0 min, 15 min, 30 min, 45 min, and 60 min. The cells were subsequently diluted by 10^5^-fold with 0.9% NaCl and inoculated onto LB agar plates for growth at 37 °C overnight. Colony forming units (CFUs) were calculated and compared to determine the survival curve.

### 2.6. Detection of ompC Expression Level

Bacteria were cultured to logarithmic stage, followed by subsequent RNA extraction with the Eastep Super Total RNA Extraction Kit (Promega Co., Madison, WI, USA). The 16s rDNA was used as the reference gene. For Quantitative Real-Time PCR (qRT-PCR) to detect the expression level of ompC, the SYBR Green Pro Taq HS Qpcr Kit III (Accurate Biology, Changsha, Hunan, China) was used. The primers used in qRT-PCR are detailed in Table S2.

### 2.7. Molecular Dynamics and Docking

A high-resolution crystal structure of *E. coli* OmpC (PDB ID: 2J1N) was used as the starting structure for molecular dynamics [28]. CHARMM-GUI was used to construct a lipid bilayer composed of 1-palmitoyl-2-oleoylphosphatidylcholine (POPC) [29]. Trimeric OmpC was then assembled into the lipid bilayer, with the addition of water, K^+^, and Cl^−^ to construct the initial system. Molecular dynamics were performed with GROMACS with the AMBER19SB force field. Simulation took place for 100 ns. The kinetic energy and potential energy were extracted within 100 ns during the simulation process to observe the changes over time in the simulation. OmpC A226D was analyzed with the same method. Molecular docking was performed with Autodock 4.2.6 [30].

### 2.8. Statistics

Comparison of MICs and gene expression levels was performed with a two-tailed *t*-test. Comparison of growth curves was performed with two-way ANOVA.

## 3. Results

### 3.1. Identification of an ompC A226D Mutation

Mutation in the *ompC* gene was identified in *E. coli* strains whose whole genomic sequences were available on Genbank. With homologous search in the Genbank database, a total of 50 strains were found to carry the OmpC_A226D_ mutation (Table S3). Thirty of these 50 strains were found to be human-origin *E. coli* strains, suggesting the clinical relevance of this mutation. Considering the involvement of OmpC in antimicrobial resistance [19,20], further work was carried out on the changes of antibiotic resistance resulting from this mutation.

### 3.2. OmpC A226D Mutation Leads to Increase of β-Lactam Susceptibility

**Figure 1:**
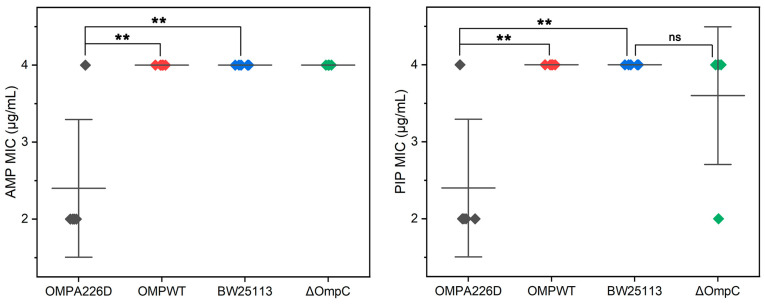
MIC values of *E. coli* strains. OMPA226D (black), *E. coli* OMP_A226D_; OMPWT (red), *E. coli* OMP_WT_; BW25113 (blue), *E. coli* BW25113. ∆OmpC (green), *E. coli* BW25113 ∆*ompC*. **, *p* < 0.01; ns, no statistical difference. Five parallels were performed in each group. The horizontal lines represent the average MIC value, vertical lines represent the SDs of the MIC averages.

The MICs of *E. coli* OMP_A226D_, *E. coli* OMP_WT_, *E. coli* BW25113 and *E. coli* BW25113 Δ*ompC* were measured for AMP and PIP (Figure 1). It can be observed that the absence of *ompC* did not affect the bacteria’s resistance to AMP and PIP, whereas the A226D mutation led to a significant decrease in MICs to both β-lactams by approximately two-fold, across five replicates.

**Figure 2:**
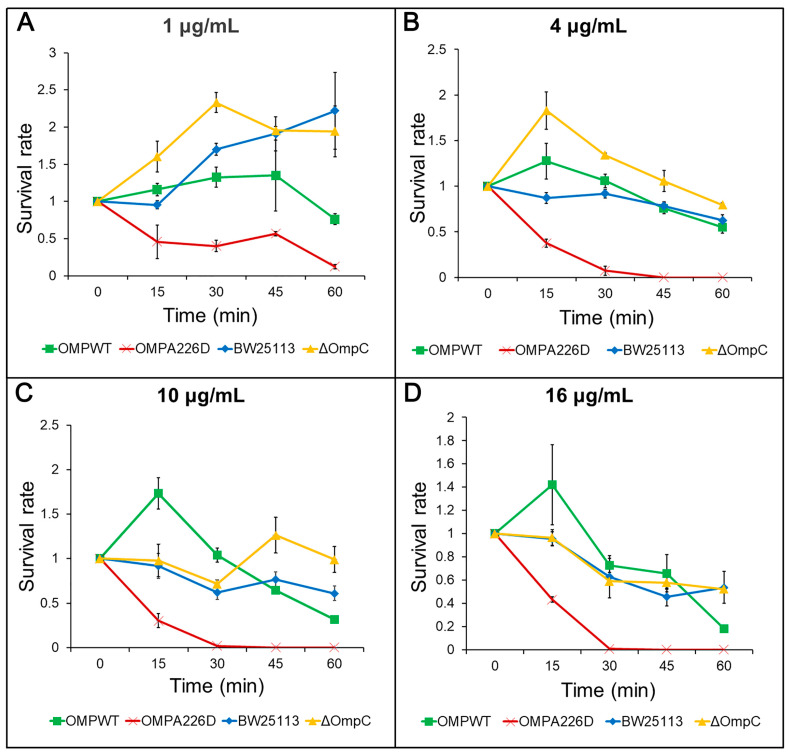
Survival assays for AMP. OMPA226D, *E. coli* OMP_A226D_; OMPWT, *E. coli* OMP_WT_; BW25113, *E. coli* BW25113. ∆OmpC, *E. coli* BW25113 ∆*ompC*. Error bars indicate standard deviations. Survival rate is the ratio of CFU numbers of the measured time point to time zero. (**A**), 1 μg/mL AMP; (**B**), 4 μg/mL AMP; (**C**), 10 μg/mL AMP; (**D**), 16 μg/mL AMP.

**Figure 3:**
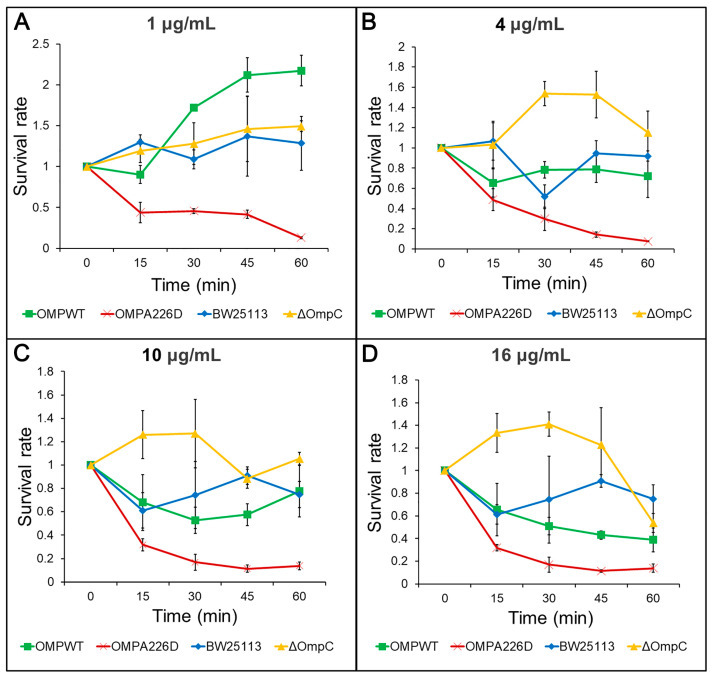
Survival assays for PIP. OMPA226D, *E. coli* OMP_A226D_; OMPWT, *E. coli* OMP_WT_; BW25113, *E. coli* BW25113. ∆OmpC, *E. coli* BW25113 ∆*ompC*. Survival rate is the ratio of CFU numbers of the measured time point to time zero. (**A**), 1 μg/mL PIP; (**B**), 4 μg/mL PIP; (**C**), 10 μg/mL PIP; (**D**), 16 μg/mL PIP.

The increase of β-lactam susceptibility following OmpC_A226D_ mutation was further confirmed with survival assays. With this more sensitive method, the *E. coli* OMP_A226D_ strain had a significantly lower tolerance to both AMP (Figure 2) and PIP (Figure 3) than *E. coli* OMP_WT_ (*p* < 0.01) and *E. coli* BW25113 (*p* < 0.01). Both the MIC measurements and survival assays confirmed that OmpC_A226D_ mutation led to increased β-lactam susceptibility. Despite showing no changes in MIC, the strain lacking OmpC exhibited superior growth compared to the *E. coli* BW25113 and *E. coli* OMP_WT_ strains when subjected to PIP and low concentrations of AMP. This observation highlights the role of OmpC in facilitating the transport of AMP and PIP.

**Figure 4:**
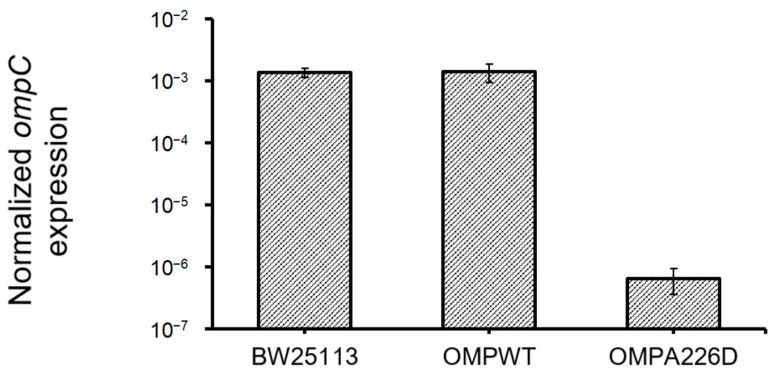
Expression *ompC* levels in all strains. OMPA226D, *E. coli* OMP_A226D_; OMPWT, *E. coli* OMP_WT_; BW25113, *E. coli* BW25113. Error bars indicate standard deviations. Expression levels were normalized with 16S rDNA levels.

The increase of β-lactam susceptibility of OmpC_A226D_ mutation suggests the increased efficiency of OmpC in the transportation of β-lactam molecules. Whether this is caused by increased OmpC transcription was analyzed by quantifying and comparing *ompC* levels in *E. coli* OMP_A226D_, *E. coli* OMP_WT_, and *E. coli* BW25113 strains (Figure 4). No significant difference was found between *ompC* levels of *E. coli* OMP_WT_ and *E. coli* BW25113 (*p* = 0.84). However, surprisingly, significantly decreased *ompC* levels were found in *E. coli* OMP_A226D_ than in *E. coli* OMP_WT_ (*p* = 6.85 × 10^−8^) and *E. coli* BW25113 (*p* = 7.98 × 10^−12^), by over 2000 fold. This strong reduction of *ompC* level, in combination with overall decreased MIC value of β-lactam antibiotics, suggests the OmpC_A226D_ mutation indeed led to significantly and substantially increased β-lactam susceptibility.

### 3.3. Structural Changes Resulting from A226D Mutation

**Figure 5:**
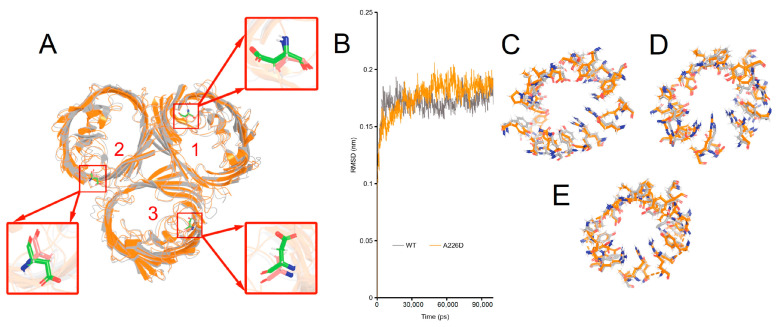
Simulated structures of OmpC wildtype and A226D proteins. Panel (**A**), alignment of wildtype and A226D OmpC proteins, numbers indicate the pore number. Panel (**B**), RMSD curves of the simulation. WT, OmpC; A226D, OmpC_A226D_. Panel (**C**), alignment of key residues in tunnel of pore 1. Panel (**D**), alignment of key residues in tunnel of pore 2. Panel (**E**), alignment of key residues in tunnel of pore 3. Gray color indicates wildtype OmpC. Orange color indicates OmpC_A226D_ mutant.

The structural basis underlying changed β-lactam susceptibility in *E. coli* OMP_A226D_ was investigated by molecular dynamics of OmpC_A226D_ in a membrane system as a previously determined trimer for wildtype OmpC [28]. In molecular dynamics, the RMSD (Root Mean Square Deviation) parameter was used to assess the structural variances between the mutant OMP and the wildtype OMP. A higher RMSD value indicates a more significant disparity between the target molecule and the reference molecule, and vice versa. Only minimal changes in the structure of OmpC_A226D_ were observed (Figure 5A, RMSD = 1.022 Å). Simulation of both the OmpC and the OmpC_A226D_ structures quickly led to stabilization (Figure 5B). Comparison of the key residues involved in the formation of the central tunnel of the three pores as indicated in Figure 5A was performed (Figure 5C–E), leading to the finding that only small changes in the positions of the key residues were present. All this evidence suggests that A226D mutation did not lead to significant changes in the structure of OmpC.

**Figure 6:**
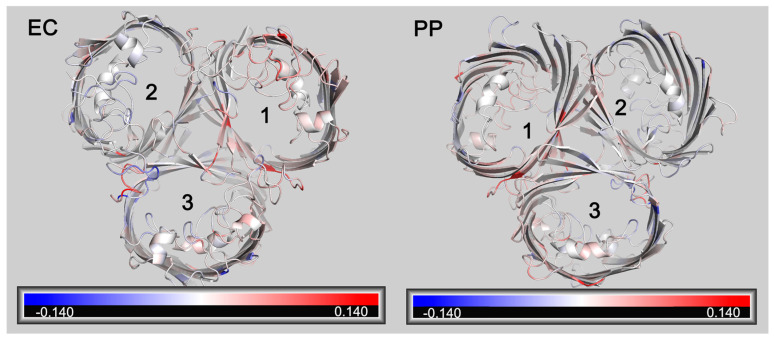
Changes of RMSF following A226D mutation. Red color indicates increased RMSF (in nm). Blue color indicates decreased RMSF (in nm). EC, view from the extracellular side. PP, view from the periplasmic side. The numbers 1,2, 3 represent pore 1, pore 2, and pore 3.

RMSF (Root Mean Square Fluctuation) is also a crucial parameter in molecular dynamics. It calculates variations in atomic motion throughout the simulation and characterizes amino acid flexibility. A further analysis of the RMSF of OmpC and OmpC_A226D_ showed that a generally increased RMSF was observed with OmpC_A226D_ (Figure 6), suggesting increased flexibility. Particularly, the extracellular loops had significantly increased RMSF for pore 2 and pore 3, and the alpha helix in the tunnel that plays a key role in tunnel formation has increased RMSF. These calculations suggest that increased flexibility that can better facilitate substrate transport plays a role in increased β-lactam susceptibility following A226D mutation.

**Figure 7:**
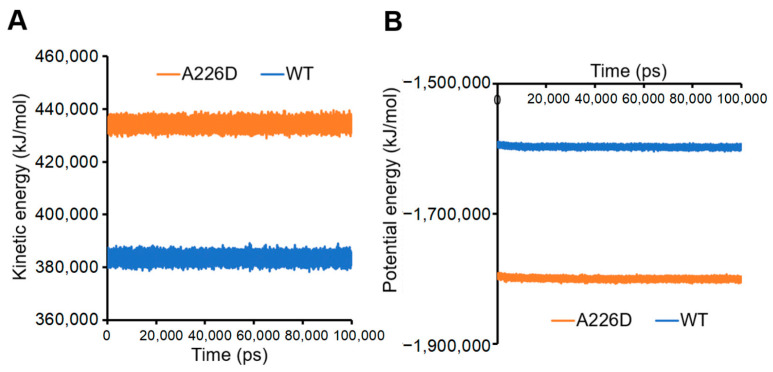
Kinetic and potential energies of simulated systems. Panel (**A**), kinetic energy. Panel (**B**), potential energy. A226D, OmpC A226D mutant. WT, wildtype OmpC.

Calculation of kinetic and potential energies in the system led to consistent conclusions. A226D mutation led to increased kinetic energy, suggesting improved flexibility and movement (Figure 7A). On the other hand, A226D mutation led to decreased potential energy, suggesting decreased interactions that accompany increased flexibility.

The above calculations led to a consistent conclusion that the A226D mutation in OmpC led to increased flexibility that may facilitate transport of β-lactams, thereby leading to increased susceptibility. Further docking analysis of OmpC with AMP did not find significant changes in binding energies between OmpC wildtype and A226D (Table 1). However, weaker binding with PIP was found between OmpC_A226D_ mutant than OmpC wildtype protein (Table 1). This finding suggests a second mechanism by which the A226D mutation leads to increased β-lactam susceptibility: by reducing interactions between OmpC and PIP, thereby allowing easier penetration of the outer membrane.

## 4. Discussion

The key finding of this work is the identification of a mutation in OmpC that can affect β-lactam susceptibility in *E. coli*. No OmpC mutation that led to antibiotic resistance was documented in the Reference Gene Catalog of NCBI, although reports on OmpC mutations that influence antibiotic susceptibility were available [31]. Like in this report, very little difference was found in the overall structure following OmpC mutation. To investigate this phenomenon further, molecular dynamics simulations were performed on both the OmpC_A226D_ and wildtype OmpC. With further molecular dynamics, we found that overall increased protein flexibility, along with consistent increased kinetic energy and decreased potential energy, could explain why the A226D mutation can lead to increased β-lactam influx.

The mutation picked for study in this work is a naturally occurring mutation that is found mostly in human-related *E. coli* strains. Because *E. coli* is a common clinical pathogen, this makes the mutation more clinically relevant: it is not an artificial mutation that is generated in the lab, although any mutation generated in the lab is also likely to occur in nature. We believe the finding of such a mutation that influences antibiotic susceptibility is important not just in theoretical aspects, but also in practical aspects. For instance, finding the presence of such a mutation in an *E. coli* strain suggests it is less likely antibiotic resistant. On the other hand, it is possible to design new drugs that target these important amino acid sites to specifically repress higher resistance strains. In combination with β-lactams, these new drugs may improve treatment efficiencies because both high resistance and low resistance strains were covered. The aforementioned methods can be utilized for clinical screening or to introduce innovative strategies for clinical treatment.

The expression level of OmpC_A226D_ was observed to be low; however, it still exhibited reduced sensitivity to β-lactam antibiotics. This suggests that the OmpC had higher permeability and weaker antibiotic binding affinity. The increase of antibiotic permeation caused by A226D mutation in OmpC was able to offset the decreased expression of OmpC in the mutation strain, which sums up to increased antibiotic influx. Therefore, the decrease of *ompC* expression in the OmpC_A226D_-expressing strain is consistent with increased antibiotic uptake by the mutant-carrying strain. It needs to be noted that the uptake of environmental chemicals by OMPs is non-specific. These proteins do not necessarily have to bind to a molecule for uptake. Therefore, interactions between small molecules and OMPs may not benefit the uptake of molecules, but on the other hand may hinder release of these molecules to the periplasm. Therefore, the decreased binding energies of OmpC_A226D_ mutant with PIP can also explain the increase of susceptibility, because the molecules can flow through OmpC pores more freely.

The mutation found in this work leads to increased antibiotic susceptibility rather than antibiotic resistance. This does not reduce its significance as molecular dynamics with this system revealed that protein flexibility and antibiotic binding may be involved in tolerance to antibiotics. If proteins evolve towards the other direction, i.e., decreased flexibility and increased binding with antibiotics, they will likely increase antibiotic resistance. The finding of such a mechanism itself improves our understanding of how cells may have tools in their toolbox to fight against antibiotic pressure.

## 5. Conclusions

In this work, a novel mutation in OmpC that increases β-lactam susceptibility was found in *E. coli*. Molecular dynamics revealed that this mutation leads to increased flexibility and decreased binding with piperacillin, which are likely the reasons for improved antibiotic uptake and susceptibility. This work represents one of the few studies that identify the relationship between OmpC mutation and antibiotic susceptibility, which may lead to further understanding of how bacteria can develop antibiotic resistance.

## Figures and Tables

**Table 1:** Docking energies of the best model calculated with Autodock.

Pores	A226D	WT
AMP		
Pore 1	−8.63	−8.8
Pore 2	−7.96	−8.56
Pore 3	−8.93	−8.03
PIP		
Pore 1	−7.17	−9.69
Pore 2	−7.77	−11.31
Pore 3	−6.64	−9.64

## Data Availability

Data is contained within the article and Supplementary Material.

## Supplementary Materials

The following supporting information can be downloaded at: https://www.mdpi.com/article/10.3390/biology13080600/s1, Table S1: Primers used in plasmid construction; Table S2: Primers used in qPCR; Table S3: OmpC A226D-carrying *E. coli* strains.
