# Supplementary material for: The A226D Mutation of OmpC Leads to Increased Susceptibility to β-Lactam Antibiotics in Escherichia coli
Source: Biology (Basel). 2024 Aug 9;13(8):600. doi: 10.3390/biology13080600 (PMC11351675; doi:10.3390/biology13080600)
Supplement: Supplementary file 1 [file biology-13-00600-s001.zip › biology-3102887-supplementary.pdf]

Table S1. Primers used in plasmid construction. Underlines indicate the segments that are homologous to other fragments, while the gray shading highlights the codon following the mutation introduced in the primer.

Primer name	Sequence (5' to 3')	Coding genes	Purpose
ompC-F	GTAAGCCAGTATACACTCCG GTTCCCTTGCATTTACA	*ompC*	Construction of pACYC-$OmpC_{WT}$
ompC-R	GACTGCGCTCCTCGCGCTAG TTAGAACTGGTAAACCAGAC		
pACYC-F	GTCTGGTTTACCAGTTCTAAC TAGCGCGAGGAGCGCAGTC	The whole plasmid of pACYC-spt	
pACYC-R	AAATGTAAATGCAAGGGAAC CGGAGTGTATACTGGCTTAC		
A226D-F	GATCATCAGTACGTTTGGAG CTGG	The whole plasmid of pACYC-spt (containing the mutation A226D)	Construction of pACYC-$OmpC_{A226D}$
A226D-R	AACGTACTGATGATCAGAAC ACCGC		

Table S2. Primers used in qPCR

Primer name	Sequence (5' to 3')	Coding genes	Purpose
qompC-F	AGTTGCGTTGTAGGTCTGGG	A part of *ompC*	For qPCR of *ompC* genes
qompC-R	TTACATCGGTAACGGCGACC		
q16S-F	CCCAGATGGGATTAGCTTGT	A part of 16S rDNA	Housekeeping gene, for normalization
q16S-R	TCTGGACCGTGTCTCAGTTC		

Table S3. OmpC A226D-carrying *E. coli* strains

Accession	Host
NC_013364.1	human
NC_007946.1	human
NZ_GG657384.1	human
NZ_GG773036.1	human
NZ_AJVT01000301.1	cow
NZ_ANYQ01000035.1	human
NZ_AIAT01000356.1	human
NZ_JHKX01000090.1	human
NZ_JHOE01000020.1	unknown
NZ_JHMS01000024.1	unknown
NZ_JWEG01000191.1	human
NZ_LPXY01000181.1	cow
NZ_CP015229.1	food
NZ_NGBR01000007.1	chicken
NZ_NONM01000002.1	cow
NZ_NXNX01000386.1	human
NZ_PGHG01000001.1	human
NZ_JZOL01000070.1	animal
NZ_BGQM01000004.1	human
NZ_BGTD01000007.1	human
NZ_CP031134.1	human
NZ_QWNF01000262.1	human
NZ_AP018802.1	cow
NZ_SAEL01000081.1	pig
NZ_CP037943.1	human
NZ_BFGQ01000010.1	human
NZ_WSVN01000001.1	treated wastewater
NZ_WTUQ01000005.1	unknown
NZ_JABBJO010000021.1	human
NZ_JABBFN010000020.1	human
NZ_CP049967.1	human
NZ_JACUWB010000001.1	human
NZ_JADDSC010000380.1	human
NZ_JADNAK010000003.1	human
NZ_CP078580.1	food
NZ_CP078582.1	human
NZ_JAIQZL010000008.1	human
NZ_SQOW01000003.1	human
NZ_JAGMOS010000003.1	chicken
NZ_CP103570.1	human
NZ_JANDAF010000001.1	human
NZ_CP123273.1	poultry egg

NZ_CP123276.1	poultry
NZ_CP125369.1	poultry egg
NZ_CP125621.1	poultry egg
NZ_SSIW01000001.1	unknown
NZ_WAIF01000022.1	human
NZ_JAVDCX010000001.1	deer
NZ_UWXG01000001.1	human
NZ_CAJZOT010000003.1	human
